# Active Arm Swing During Running Improves Rotational Stability of the Upper Body and Metabolic Energy Efficiency

**DOI:** 10.1007/s10439-025-03688-0

**Published:** 2025-02-03

**Authors:** Young-Jun Koo, Naomichi Ogihara, Seungbum Koo

**Affiliations:** 1 Artificial Intelligence Computing Research Laboratory, Electronics and Telecommunications Research Institute, Daejeon, Republic of Korea; 2 The University of Tokyo, Bunkyo City, Tokyo Japan; 3 Department of Mechanical Engineering, Korea Advanced Institute of Science and Technology, 291 Daehak-ro, Yuseong-gu, Daejeon, 34141 Republic of Korea

**Keywords:** Arm swing, Metabolic energy, Musculoskeletal model, Forward dynamics

## Abstract

**Purpose:**

The kinematic benefits of arm swing during running for upper body stability have been previously investigated, while its role in metabolic energy efficiency remains controversial. To address this, this study aimed to test the hypothesis that active arm swing during running reduces both torso angular motion around the longitudinal axis and metabolic energy consumption.

**Methods:**

We employed forward dynamics musculoskeletal running simulations with different arm conditions to investigate the hypothesis. Full-body musculoskeletal running models, incorporating 150 muscles, were developed using artificial neural network-based running controllers. Three arm conditions were simulated using the running models and controllers: active arm swing, passive arm swing, and fixed arms.

**Results:**

Our results revealed that the active arm swing model demonstrated the lowest total metabolic energy consumption per traveling distance. The costs of transport were 5.52, 5.73, and 5.82 J/kg-m for active, passive, and fixed arm models, respectively. Interestingly, while metabolic energy consumption in the upper limb muscles was higher during active arm swing, the total energy consumption was lower. Additionally, the longitudinal rotation of the torso was minimal in the active arm swing condition.

**Conclusion:**

These findings support our hypothesis, demonstrating that active arm swing during running reduces the angular motion of the torso and the metabolic energy consumption. This study provides evidence that arm swing during running is performed actively as an energy-saving mechanism. These results contribute to understanding of running biomechanics and may have implications for performance optimization in sports and rehabilitation settings.

**Supplementary Information:**

The online version contains supplementary material available at 10.1007/s10439-025-03688-0.

## Introduction

Humans naturally swing their arms during bipedal movements, such as walking and running. Arm swinging during walking has been shown to be a combination of passive and active motions through electromyography-based experimental studies [13, 24] and dynamics-based simulation studies [8]. The effects of arm swinging during walking has been extensively investigated. Arm swinging during walking contributes to decreased metabolic energy expenditure [8, 9, 30, 40] and affects the body’s center of mass in the vertical direction, which is frequently depicted as a pendular movement [12, 17]. Additionally, during walking, the out-of-phase arm swinging relative to leg movements help reduce the vertical whole-body angular momentum during walking [7, 15].

Running has different dynamics from walking. Arm swinging during running appears to be dominated by active movement [22]. The biomechanical benefits of arm movement patterns during running have been extensively investigated. Arellano and Kram [3] found that the arm swing helps increase mediolateral stability to compensate for a narrow step width during running compared to walking, resulting in reduced energy costs. Hinrichs [16] suggested that arm swing reduces metabolic costs by counterbalancing the angular momentum of lower limbs. Other studies have reported that arm swing plays an important role in contributing to the vertical oscillation of the body’s center of mass [1] and vertical ground reaction forces [25].

However, conflicting findings exist in the literature regarding the impact of arm swing on energy costs. Egbuonu et al. [11] and Pontzer et al. [33] reported that running without an arm swing does not increase energy cost. Conversely, other studies have found that eliminating arm swing during running increases metabolic energy as measured by oxygen consumption and carbon dioxide production [4, 44]. The increased metabolic energy expenditure during running without arm swing could be attributed to the additional effort required to maintain the unnatural posture of keeping the hands on the head or chest. This posture likely demands greater muscle activity, leading to higher energy expenditure compared to running with natural arm movements [27, 33, 39]. The conflicting results from previous studies regarding the role and mechanical consequences of arm swinging during running in terms of metabolic energy efficiency have not been well understood [27].

Musculoskeletal dynamics simulations have been utilized to compare energy consumption [5] and ground reaction force during walking under different arm conditions [8]. Forward-dynamics-based musculoskeletal simulations have been employed to implement predictive simulations under various locomotion or musculoskeletal conditions [29, 42, 43]. The implementation of predictive simulations requires control strategies for skeletal muscles, such as central-pattern generator and muscle reflexes, to simulate gait motion using a musculoskeletal model [2, 37]. Aoi et al. [2] implemented adaptive gait simulations of a two-dimensional musculoskeletal model under different gait conditions, such as perturbing external forces or various sloped terrains, using a proposed central-pattern generator-based model. Building upon this work, the effect of arm swing on running kinematics and energy expenditure can be analyzed by developing a musculoskeletal running model under different arm conditions in a three-dimensional forward dynamics-based environment. Recent advancements in artificial intelligence have demonstrated the potential for obtaining controllers for gait or other movements of complex dynamic systems [26]. Deep reinforcement learning (RL) offers the advantage of identifying control strategies for high-dimensional dynamic systems [35]. In the field of biomechanics, RL has been studied to learn human locomotion behaviors [20, 31]. The deep RL method can be applied to obtain a running controller for a complex full-body musculoskeletal model, both with or without arm swing, allowing for a comprehensive comparison of these conditions.

The aims of this study were twofold: (1) to develop a full-body musculoskeletal running model using artificial neural network-based running controllers, and (2) to test the hypothesis that active arm swing during running reduces the angular motion of the torso around the longitudinal axis and metabolic energy consumption of body skeletal muscles. We posit that arm swing during running may counterbalance the angular momentum generated by both the braking forces when landing and the active forces during the push-off phase of running, thereby reducing the effort required by body muscles to maintain stability and enhancing running performance. To test this hypothesis, we developed a physiological full-body musculoskeletal model incorporating 150 muscles and running controllers. Three distinct arm conditions were examined through running simulations: (1) active arm swing, (2) arms fixed to the torso, and (3) passive arm swing. Using forward dynamic musculoskeletal running simulations, we analyzed changes in angular motion with respect to the longitudinal axis and metabolic energy consumption under these different arm swing conditions. This comprehensive approach allows for a detailed examination of the biomechanical and energetic effects of arm swing during running, contributing to our understanding of human locomotion efficiency and potentially informing applications in fields, such as sports science, rehabilitation, and robotics.

## Materials and Methods

### Data Acquisition

This study was approved by the Institutional Review Board of Korea Advanced Institute of Science and Technology. All methods were performed in accordance with the relevant guidelines and regulations. A healthy young adult (age 23 years, weight 65.4 kg, and height 171.2 cm) without a history of lower limb injury in the last five years was recruited for this study after obtaining informed consent. The participant, as a recreational runner, regularly engaged in non-competitive running activities. The subject had experience maintaining a consistent running routine as a hobby, though not at an elite or competitive level. Reflective body markers were attached to the participants according to the Plug-in Gait full-body marker set protocol of the Vicon motion capture system (Oxford, UK). A treadmill equipped with embedded force plates (Bertec TM-09 instrumented split-belt with 2.6 kW motor, Bertec Corporation, Columbus, OH) was used. Prior to data collection, the participant underwent a familiarization period, walking and running for more than 10 minutes until he could maintain a natural gait. Following the practice session, the participant ran at 12 km/h for three minutes to record the data. During this running trial, the trajectories of the body markers were recorded using the motion capture system, while the ground reaction forces (GRF) were simultaneously measured by the force plates embedded in the treadmill. A cycle of running data, from right heel strike to the next right heel strike, was extracted from the middle of the three-minute recording, during which kinematics remained stable, with foot placement variation below 20 mm for five consecutive cycles. This data acquisition protocol facilitated the collection of kinematic and kinetic data during running, which was subsequently used for analysis and modeling.

### Musculoskeletal Model

**Fig. 1:**
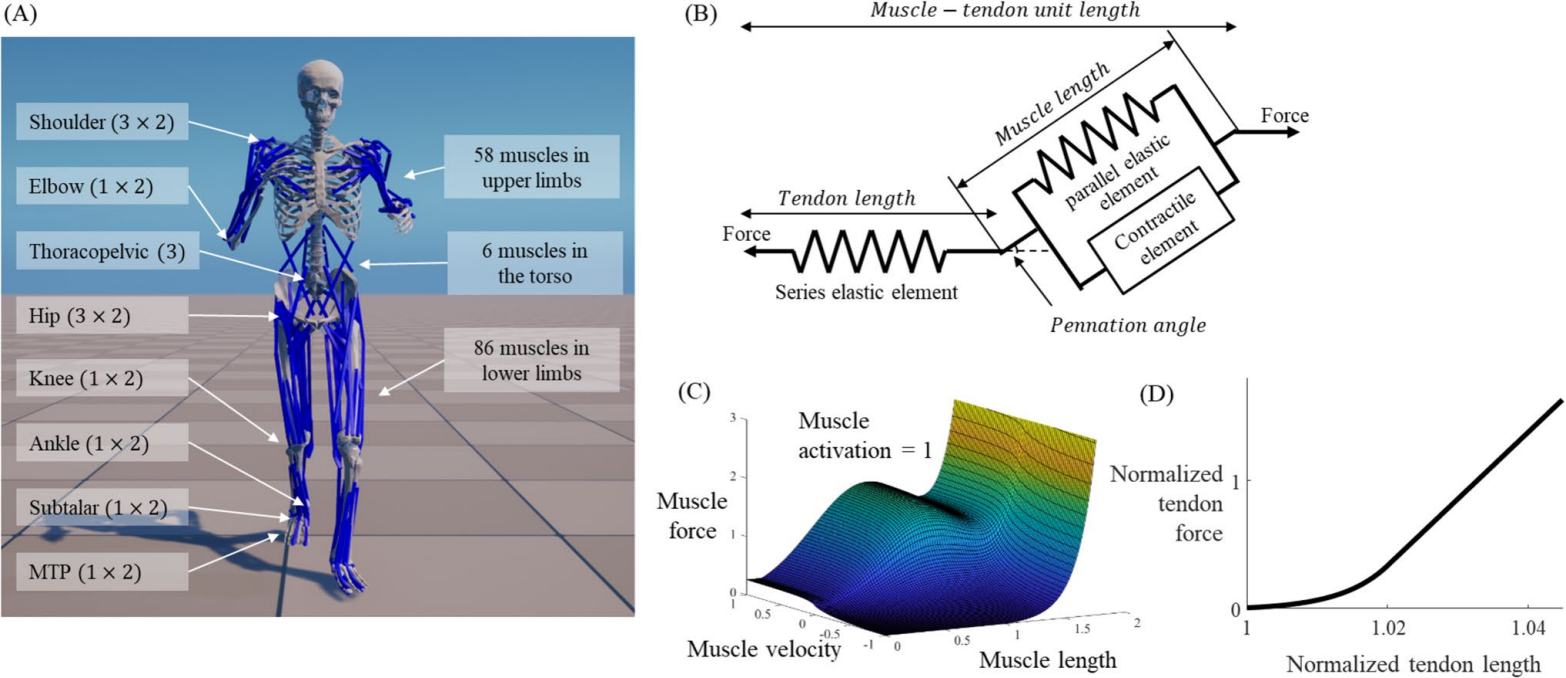
**A** A full-body musculoskeletal model with 150 muscles, **B** a muscle-tendon model, **C** a force-length-velocity relationship of the muscle model, and **D** a force-length relationship of the tendon model

A three-dimensional full-body musculoskeletal model with 25 degrees of freedom excluding six degrees of freedom for the pelvis root body and 150 muscles was constructed to implement dynamic gait simulation in the RaiSim dynamics environment [19]. Skeletal geometries, including mass, inertia, and joint information of the musculoskeletal model, were obtained from published and open musculoskeletal model [34]. The model was modified to include a total of 25 degrees of freedom, comprising thoracopelvic, shoulder, and hip spherical joints, as well as elbow, knee, ankle, subtalar, and metatarsophalangeal revolute joints (Fig. 1A). A custom Hill-type muscle model with an activation dynamics component was developed to provide muscle force for given muscle length and velocity from muscle excitation signals, using young adult muscle parameters and equations in a previous study [38]. This custom model consisted of a series combination of the tendon as a passive element and the muscle as both passive and active elements (Fig. 1B–D). Model parameters for each muscle-tendon unit, including the force-length relationship of the tendon and the force-length-velocity relationship of the muscle, were derived from the Gait2392 and UpperExtremity models [18] in OpenSim. Muscle attachment positions and activation dynamics parameters were obtained from these models. All joints in the musculoskeletal model were actuated solely by muscle models. Foot-ground contact models were implemented using a sphere in the calcaneus and two spheres in the toe for both legs. The contact module of the RaiSim dynamics solver was employed to estimate contact and friction forces [19].

### Reinforcement Learning for a Reference Running Controller

**Fig. 2:**
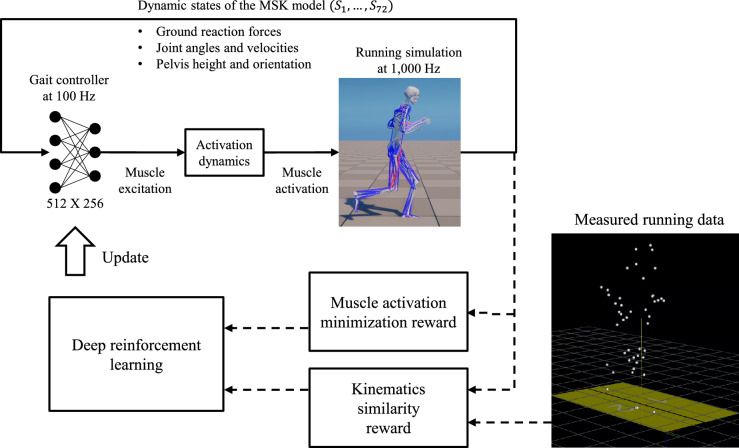
A forward dynamics environment for musculoskeletal running simulations and a deep reinforcement environment using a measured running motion (solid line: forward dynamics simulation; dashed line: deep reinforcement learning)

A deep neural network-based reference running controller that actively swung arms was obtained using the deep RL method (Fig. 2). This controller generated muscle excitation signals for 150 skeletal muscles based on feedback from the dynamic states of the musculoskeletal model to mimic human running kinematics. The deep RL method optimized the neural network parameters of the controller to determine muscle excitation signals that maximized a reward or training objective. The reward was calculated as the sum of motion tracking and muscle activation scores. The motion-tracking score, indicating how well the musculoskeletal model mimicked human running kinematics, was obtained by calculating four differences: joint angles, joint angular velocities, the position of the body’s center of mass, and the positions of both feet, hands, and the head. The equations and coefficients for calculating the reward were derived from our previous study [20]. The muscle activation score increased by decreasing the sum of the squared activations of the 150 muscles encouraging the controller to track human running kinematics with minimum use of skeletal muscles.

Human running kinematics were prepared using the model scaling and inverse kinematics functions of OpenSim [10]. The running controller’s input comprised 72 dynamic state values, including the orientation of the pelvis (3), its rotational and translational velocities (6), its height (1), the angular position of each joint (25), its angular velocity (25), ground reaction forces (6), and the center of pressure (6) of each foot with respect to the pelvis frame. The proximal policy optimization algorithm [36] was employed for the deep RL training of the human model [20]. Each update of the running controller during deep RL used samples of 80,000 from 4.0 second simulations across 200 environments. After 80,000 updates (approximately 72 h of training), we obtained a running controller capable of tracking the reference running kinematics.

### Reinforcement Learning for Different Arm Condition Controller

**Fig. 3:**
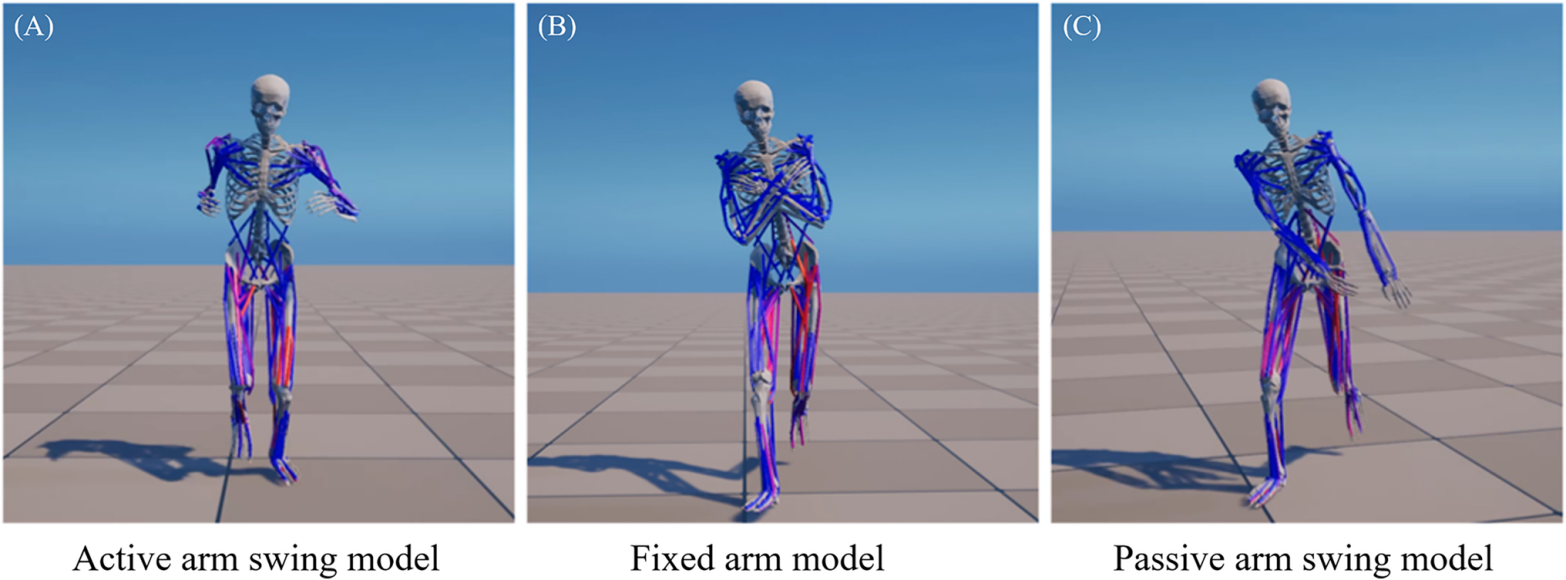
Running simulations of **A** active arm swing, **B** fixed arm, and **C** passive arm swing conditions using musculoskeletal models and neural network-based running controllers

The reference running controller was adjusted to produce two additional running controllers with two different arm conditions (Fig. 3): passively swinging arms with no upper limb muscle activation, and arms crossed and fixed to the chest. The fixed arm position at the chest was chosen based on prior studies [3, 33], which reported conflicting results regarding the impact of arm swing on metabolic cost during running. These additional controllers were obtained by modifying the reward function and the musculoskeletal model, followed by retraining.

For the fixed arm controller, the shoulder and elbow joints were constrained to maintain a crossed-arm position at the chest. The reward function for training this controller excluded differences in upper limb kinematics between human running and musculoskeletal model kinematics. For the passive arm swing controller, the shoulder and elbow joints remained unmodified, but the 58 upper limb muscles were deactivated. Similarly, the reward function for this controller also excluded differences in the upper limb kinematics. After these modifications to the reward functions and musculoskeletal models, each controller underwent retraining. This process involved 40,000 updates over approximately 26 h, using 80,000 samples for each update. This retraining ensured that the controllers were optimized for their specific arm conditions while maintaining the overall running kinematics.

### Analysis of the Effect of Arm Swing During Running

Forward dynamics-based musculoskeletal running simulations were performed for 20 s under three different arm conditions: active arm swing, passive arm swing, and fixed arms. Due to the nature of forward dynamics simulation for continuous running motions, consecutive cycles are dynamically connected, leading to small variations, such as asymmetry between cycles. To account for these variations, simulation results, including energy consumption and kinematics, were averaged over the 20-second simulation. Total metabolic energy costs during the simulations were estimated based on the mechanical work of skeletal muscles and basal energy rate, following the methods described by Margaria [23] and Ogihara et al. [28]. A basal energy rate coefficient of 1.2 W/kg for humans was used according to a reference study [41]. The basal rate, determined by the total mass of the musculoskeletal models, remained constant across all arm conditions. For the mechanical work of the skeletal muscles, different weight coefficients were applied: 0.25 for positive work and − 1.2 for negative work, as used in Margaria [23] and Ogihara et al. [28]. Torso kinematics were compared across the different arm conditions to analyze the effect of arm swing on the rotational stability of the torso during running.

To validate the musculoskeletal model, simulated GRFs under the three arm swing conditions were qualitatively compared to experimentally measured GRFs. Both the measured and simulated GRFs were normalized to the stance phase of the gait cycle for direct comparison, allowing for an assessment of the running model and simulation validity.

## Results

The total metabolic energy consumption per traveling distance varied across the three arm swing conditions, with the active arm swing controller demonstrating the lowest energy cost. The costs of transport were 5.52, 5.82, and 5.73 J/kg-m for active arm swing model (AASM), fixed arm model (FAM), and passive arm swing model (PASM), respectively. While the PASM and FAM did not utilize upper limb muscles, resulting in no metabolic energy consumption in these areas, they showed increased metabolic energy consumption in the muscles around the torso and lower limbs compared to the AASM. The transport costs for the total body, upper limbs, torso, and lower limbs are summarized in Table 1. The basal costs per traveling distance varied slightly among the models due to differences in running speeds across the arm conditions. Specifically, the running speeds of the AASM, FAM, and PASM were 3.24, 3.11, and 3.20 m/s, respectively.

**Table 1:** Metabolic costs during musculoskeletal running simulations with different arm conditions

	Active arm swing model	Fixed arm model	Passive arm swing model
Gross metabolic costs of transport (J/kg-m)	5.52	5.82	5.73
Metabolic costs in upper limb (J/kg-m)	0.71	0.00	0.00
Metabolic costs in torso (J/kg-m)	0.55	0.71	0.79
Metabolic costs in lower limb (J/kg-m)	3.89	4.72	4.56
Basal costs of transport (J/kg-m)	0.37	0.39	0.37

**Fig. 4:**
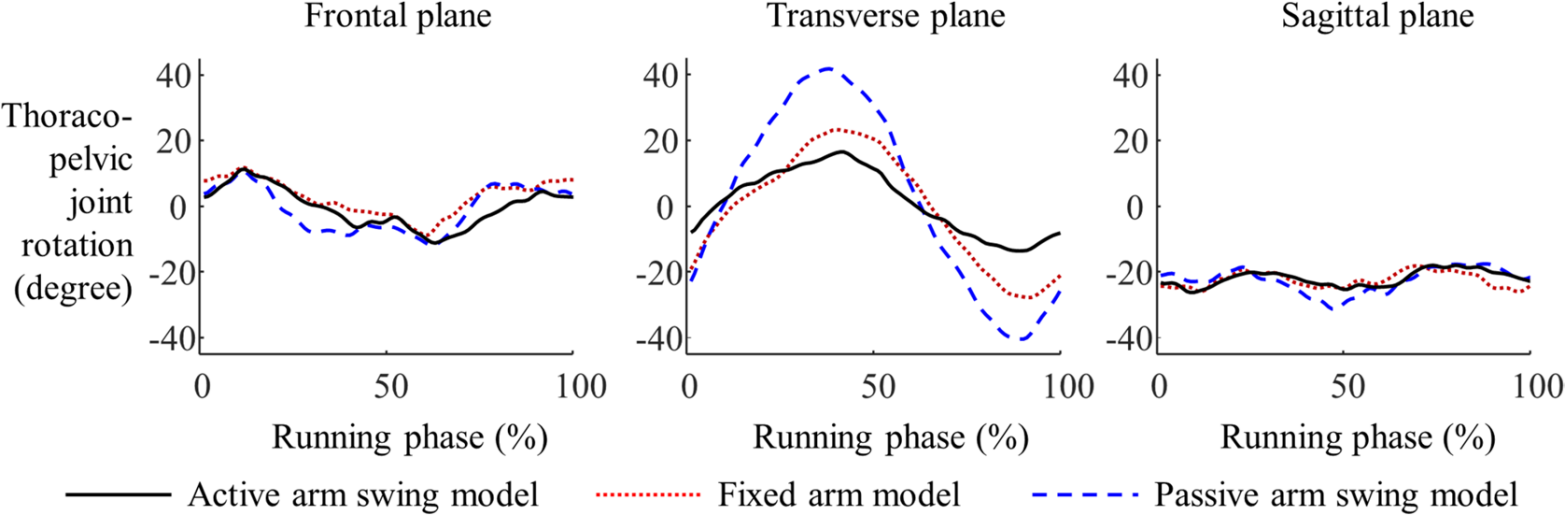
Longitudinal rotation of the torso relative to the pelvis during running simulations (black line: active arm swing model, red line: fixed arm swing model, blue line: passive arm swing model)

**Fig. 5:**
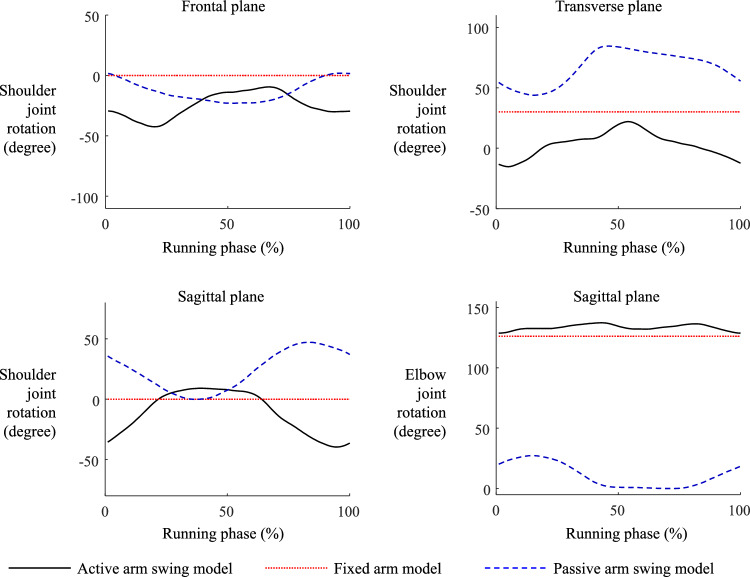
Shoulder and elbow joint rotations during running simulations (black line: active arm swing model, red line: fixed arm swing model, blue line: passive arm swing model)

The longitudinal rotation of the thorax relative to the pelvis was the smallest in the AASM group. The ranges of longitudinal rotational motion of the upper body were 30.5°, 51.1°, and 75.0° for the AASM, FAM, and PASM, respectively. Thoracopelvic joint kinematics in the sagittal and frontal planes showed no qualitative differences across the models (Fig. 4). The upper limb kinematics, represented as the phase-matched mean values of the left and right arms, qualitatively differed between the AASM and PASM, as illustrated in Fig. 5. Shoulder joint rotation in the sagittal plane revealed contrasting patterns between the AASM and PASM. Additionally, the AASM exhibited a greater elbow flexion angle compared to the PASM. These findings highlight the significant impact of different arm swing conditions on upper body rotation and upper limb movements during running.

**Fig. 6:**
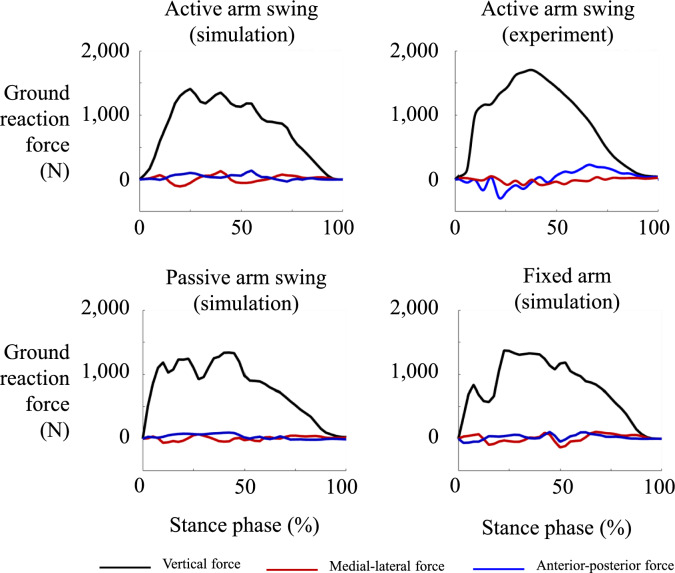
Simulated ground reaction forces (GRF) under different arm swing conditions and experimentally measured GRFs under the active arm swing condition during the stance phase of running

Figure 6 presents a comparison of experimental and simulated GRFs for each arm swing condition, including vertical, medial-lateral, and anterior-posterior components. The results indicated that the simulated GRFs closely matched the experimentally measured GRFs. The simulated vertical force closely agreed to the measured GRF values, with both similar peaks and patterns throughout the stance phase. The medial-lateral and anterior-posterior forces also exhibited a similar trend, with minor deviations observed. The vertical GRF comparison demonstrated high similarity between simulation and experimental data. While the medial-lateral and anterior-posterior forces are generally aligned, there are slight differences in magnitude and timing. The root mean squared differences of ground reaction forces between the experimental and simulated data for running with active arm swing were 123.1 N in the anterior-posterior direction, 148.5 N in the medial-lateral direction, and 250.7 N in the vertical direction.

## Discussion

This study investigated running simulations under various arm swing conditions using a physiological full-body musculoskeletal model comprising 150 muscles and running controllers. We have developed a full-body musculoskeletal running model capable of accurately simulating running dynamics, with ground reaction force patterns and magnitudes closely matching experimental data. The developed model facilitated the isolation and analysis of arm swing’s biomechanical effects on running dynamics under different arm conditions. By employing forward dynamics simulation, this study provided a comprehensive evaluation of arm swing contributions.

Our findings support the hypothesis that active arm swing during running reduces both the angular motion of the torso around the longitudinal axis and the metabolic energy consumption of the skeletal muscles. The results demonstrate that active arm swing contributes to improved metabolic energy efficiency during running, consistent with previous studies [4]. Interestingly, while metabolic energy consumption in the upper limb muscles was higher during active arm swing, the total energy consumption was lower compared to other conditions. These results suggest that arm swing during running is performed actively as an energy-saving mechanism, aligning with earlier propositions [16].

The kinematic results of the thoracopelvic joint in the transverse plane corroborate previous findings that active arm swing contributes to improved rotational stability in the torso [8, 25]. The observed lower rotation in the transverse plane of the AASM suggests that angular instabilities caused by rapid and large movements of the lower limbs may be compensated for by active arm swing [7]. In the absence of active arm swing, angular stability appears to be maintained through increased torso rotation [25]. This explains the larger range of motion observed in the thoracopelvic joints in the transverse planes of both the PASM and FAM, which likely emerges as a mechanism to maintain angular stability during running. Furthermore, arm swing during running may serve to counterbalance the angular momentum generated by the foot braking force, thereby reducing the muscular effort required to maintain body stability [14]. These findings emphasized that active arm swing plays a crucial role in reducing the compensatory demands placed on the torso, allowing for more efficient and stable running mechanics. It should also be noted that our comparisons and findings among AASM, PASM, and FAM do not contrast with the association between the increase in arm swing and the increase in torso rotation in runners with active arm swing [21].

Our study demonstrates that running with arms crossed and fixed to the chest results in an increased energy consumption of approximately 5%. This finding aligns with previous research by Arellano and Kram [3], which reported that treadmill running with arms crossed in front of the chest significantly increased net metabolic power requirements by 8% compared with running with swinging arms. In experimental settings, the elimination of arm swing by placing hands in front of the chest may lead to increased metabolic energy consumption due to the muscular effort required to maintain upper limb posture [27, 39]. However, our musculoskeletal model with fixed arm conditions did not necessitate additional effort to maintain the arms’ position, as the upper limb joints were dynamically fixed. This methodological difference allowed us to isolate and estimate the effect of restricted arm swing on metabolic energy expenditure without confounding factor of postural maintenance effort. The increase in metabolic energy consumption due to fixed arms in our study was proportionally lower than that observed in previous experimental studies. This discrepancy may be attributed to the absence of additional muscular effort for posture maintenance in our model. Pontzer et al. [33] conducted experiments under fixed arm, weighted arm, and normal arm swing conditions to manipulate the moment of inertia of the arms and upper body. The study noted that variations in moment of inertia could differ between participants, suggesting that individual characteristics might influence the results. In the fixed arm condition, significant kinematic changes were observed, including increased vertical rotation of the head, altered phase differences between the pelvis and shoulders, and changes in stride frequency. These findings suggested that participants adapted their kinematic patterns to optimize energy efficiency. However, further investigation is required to determine whether these adaptations can fully compensate for the biomechanical role of arm swing.

The kinematic results of the PASM differ from those of the AASM. This discrepancy underscores that the natural arm swing that occurring during human running cannot be passively generated but is predominantly actively controlled [22]. While previous studies have suggested that arm swing during walking represents a combination of passive and active arm motions [13, 24], our findings demonstrate that active control has a dominant effect during running. A key observation is the absence of large elbow flexion in the PASM, which is a characteristic feature of running that distinguishes it from walking [22]. This absence in the passive model further supports the notion that arm swing during running is primarily driven by active control rather than being a passive phenomenon. These results have important implications for our understanding of human locomotion biomechanics, suggesting that the neuromuscular control of arm movements during running is more complex and actively regulated than previously thought, as arm swing has been viewed as serving a passive mass damper role rather than directly contributing to metabolic efficiency [33]. These findings highlighted the necessity of further exploring the interplay between active neuromuscular control and passive biomechanical properties to better understand the functional contributions of arm swing during running. This active control may play a crucial role in optimizing energy efficiency and maintaining stability during high-speed locomotion.

Our study revealed that the arm conditions of the musculoskeletal model significantly influenced the running velocity obtained through the deep RL training. Interestingly, this variation occurred despite the consistent velocity of the reference running kinematics used in the training objective across all conditions. These findings align with previous research that has highlighted the role of arm swing in improving running performance [6, 22]. Our results demonstrate that altering conditions indeed affects running performance, corroborating the outcomes of previous experimental studies. The observed differences in running velocities across arm conditions, despite consistent reference kinematics, suggest that arm swing plays a crucial role in optimizing running mechanics.

Traditional methods of studying arm movement during running often involved physically constraining or binding the arms of participants. This approach, while proving valuable insights, had inherent limitations as it could cause discomfort to the participants and might induce psychological stress, potentially influencing study outcomes. Our study addressed these limitations by employing a novel approach using reinforcement learning-based controllers to simulate running in a forward dynamics environment [20]. This methodology offers several key advantages. The simulation-based approach effectively eliminated psychological factors that could confound results in traditional studies. This method allowed us to independently verify the impact of arm swing on running performance without the interference of psychological or comfort-related variables.

A notable limitation of this study is the higher cost of transport observed in our running models compared to human subjects. Specifically, the AASM demonstrated a cost of transport of 5.52 J/kg-m, which exceeds the 3.70 J/kg-m calculated for human running at 3.24 m/s based on previous research [32]. Pontzer’s study proposed that the cost of transport for human running could be estimated using the formula: − 0.06 × (running velocity) + 3.89. The discrepancy may be attributed to our methodology of calculating total metabolic cost as the sum of metabolic energy consumed by individual muscles [28]. The inclusion of a large number of muscles in our musculoskeletal running model might have led to an overestimation of the cost of transport. Despite this limitation, the relative changes in the cost of transport under different arm conditions closely mirrored those observed in human subjects. Notably, the 5.4% increase in the cost of transport for the FAM aligns well with previous research findings, which reported a 4 to 8% increase in metabolic costs when arm swing was restricted [3, 11, 22]. Furthermore, the comparison between experimental and simulated GRFs provided additional validation for our musculoskeletal model. The close agreement between simulated and measured GRFs underscores the robustness and accuracy of running model simulations, lending credibility to our findings despite the noted limitations in absolute cost of transport values. Our simulation model was based on a single body size and a single running controller from one experiment. However, the primary focus of our study was on three distinct arm swing conditions, emphasizing task-level differences rather than inter-individual variations. Nonetheless, future studies could extend this work by including diverse subjects to further investigate inter-individual variability.

Our findings had practical implications across several fields. In performance optimization, the model can help coaches design training programs to enhance running efficiency through active arm swing, which stabilizes upper body rotation. In rehabilitation, it provides insights for developing retraining exercises that improve stability and reduce strain for patients with motor impairments. Additionally, the model can guide the development of assistive devices, such as robotic limbs and exoskeletons, optimizing their design to replicate human stability mechanisms. These applications demonstrate the broader impact of this modeling approach beyond traditional experimental methods. Although further validation with diverse populations is necessary to confirm the robustness and accuracy of the suggested model for clinical use.

In this study, we simulated active arm swing, fixed arm, and passive arm swing during running using forward dynamics-based musculoskeletal models with novel running controllers trained using a deep RL method. Our findings demonstrate that active arm swing during running significantly improves upper body stability and enhances metabolic energy efficiency. The underlying mechanism for the reduction in energy consumption by active arm swing during running can be explained by the thoracopelvic rotation and metabolic energy consumption in the muscles around the lower limb and torso. We observed that restriction of arm swing causes angular instabilities and increases whole-body angular momentum in the direction of the longitudinal axis. This underscores the importance of arm movements in maintaining stability and efficiency during high-speed locomotion. The innovative approach employed in this study, combining musculoskeletal modeling with deep reinforcement learning, has provided valuable insights into the biomechanics and energetics of running. These findings significantly enhance our understanding of the role of arm movements during running and have potential implications for various fields, including sports science, rehabilitation, and the design of running-capable humanoid robots and prosthetic limbs.

## Supplementary Information

Below is the link to the electronic supplementary material.

Supplementary file1 (MP4 49616 KB)
